# Design High-Efficiency III–V Nanowire/Si Two-Junction Solar Cell

**DOI:** 10.1186/s11671-015-0968-2

**Published:** 2015-06-26

**Authors:** Y Wang, Y Zhang, D Zhang, S He, X Li

**Affiliations:** College of Electrical Engineering and Automation, Anhui University, Hefei, China; Key Laboratory of Material Physics, Institute of Solid State Physics, Chinese Academy of Sciences, Hefei, 230031 China

**Keywords:** Two-junction solar cells, Nanowire, Current matching, Photovoltaics

## Abstract

In this paper, we report the electrical simulation results of a proposed GaInP nanowire (NW)/Si two-junction solar cell. The NW physical dimensions are determined for optimized solar energy absorption and current matching between each subcell. Two key factors (minority carrier lifetime, surface recombination velocity) affecting power conversion efficiency (PCE) of the solar cell are highlighted, and a practical guideline to design high-efficiency two-junction solar cell is thus provided. Considering the practical surface and bulk defects in GaInP semiconductor, a promising PCE of 27.5 % can be obtained. The results depict the usefulness of integrating NWs to construct high-efficiency multi-junction III–V solar cells.

## Background

Multi-junction solar cells are constituted by series-connected multiple semiconductor layers (subcells) to convert the energy of light directly into electricity by the photovoltaic effect. By dividing the broad solar spectrum into smaller sections, the multi-junction solar cell can convert solar energy more efficiently. Till now, efficiencies exceeding 44 % have been achieved at high concentration ratios for conventional triple-junction cell cells [1]. However, the high efficiency of thin-film multi-junction solar cell is gained at the cost of increased complexity and manufacturing price. The development of high-efficiency, low-cost photovoltaic device is urgently needed.

Due to their potential to realize low-cost and high energy conversion efficiency solar cells, semiconductor nanowire array (NWA) is a topic of intense research for photovoltaic applications. Recently, many theoretical and experimental works, which are focused on the design of single III–V NWA *p*-*n* junction, are widely reported [2, 3]. Meanwhile, some axially connected nanowire two-junction cells are also theoretically designed to obtain high-efficiency solar cells [4]. Considering the fact that many kinds of III–V NWAs have been successfully prepared on hetero-substrates (such as Si) [5], the concept of multi-junction solar cell can also be used in the design of photovoltaic devices. As compared with conventional multi-junction solar cell, the integration of NWAs in photovoltaic applications has the following merits. Firstly, NWA with well-defined geometrical structures exhibits higher light absorption than their thin-film counterparts of the same thickness, due to the integration of NWA in solar cells significantly changes the mechanism of light absorption [6]. Secondly, when the top NWA cell is designed in radial *p*-*n* structure, the short collection lengths facilitate the efficient collection of photogenerated carriers, and herein, the Shockley-Quisser efficiency limit for nanowire arrays is higher than for the conventional thin film [7]. Finally, the integration of semiconductor NWA on low-cost substrates can help substantially reduce the final cost of PV fabrication.

In view of this, we have recently presented a study on two-junction III–V NWA/Si solar cell, which is consisted by III–V NWA with *p*-*n* junction on the active Si substrate. We show that outstanding light harvesting rooted from the strong light trapping and the formation of Fabry-Pérot optical cavity in the NWA enables the cell to produce high photocurrent [8]. In this paper, we further report the electrical performance via simulation, where the physical dimensions are taken by considering the condition of current matching and maximized solar energy absorption. The impact of III–V NWA quality in terms of minority carrier lifetime, surface recombination velocity (SRV) on the solar cell performance is presented, and a practical guideline to design high-efficiency III–V NWA/Si two-junction solar cell is provided.

## Methods

### Experiments

**Fig. 1:**
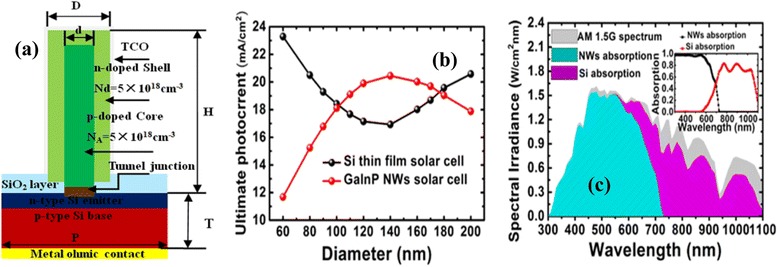
**a** Schematic diagram of the proposed III–V NWA (with core-shell structure)/Si thin-film two-junction solar cell. **b** Ultimate photocurrent of subcells for various diameters of NWs. **c** The AM1.5 solar spectrum and the calculated absorption in GaInP top subcell (with NW diameter of 100 nm) and bottom silicon cell

Figure 1a shows the schematics of the proposed two-junction solar cell, which is comprised of vertically aligned core-shell structure Ga_0.35_In_0.65_P (1.7 eV) NWs [9, 10] on active thickness (*T*) 400 μm Si (1.12 eV) substrates. By balancing the absorption and consumption of source materials, in this study, we fixed the length (*L*) of GaInP NW at 2 μm. The NWA cell was connected with Si thin-film cell by the tunnel junction which can be assumed to be perfect. The SiO_2_ layer is added between the shell of NWs and the Si substrate. Transparent conductive oxide (TCO) and metal contacts are placed at the top and bottom of the structure.

The optical properties of GaInP NWA/Si solar cells are investigated using Sentaurus Electromagnetic Wave Solver module package, a finite-difference time-domain (FDTD) full field electromagnetic simulation tool [11]. In order to simplify the calculation, the Si thin film is assumed infinite thick with no transmission loss by using a perfect match layer (PML) adjacent underneath the Si film.

To investigate the potential photovoltaic efficiency of top NWA solar cells, a coupled optoelectronic simulation is presented. Based on the position-dependent light absorption obtained by FDTD simulation, the three-dimensional (3D) carrier generation rate is obtained from the divergence of the Poynting vector. The current-voltage behaviors of the GaInP NWA subcells are calculated at three-dimensional by using the weighted optical generation profiles [12]. The electrical behaviors of the bottom Si cell are calculated at two-dimensional with Beer-Lambert absorption model while a reflection loss is adopted from the FDTD simulations. Ohmic contacts are assumed to be ideal at the front and rear surfaces of the device. The contact resistance has a minimum effect on the cell performance for resistivity of less than 0.01 Ω cm^2^. The tunnel diode connecting the subcells is assumed to be perfect. Electrical simulation takes into account the doping-dependent mobility, Auger, radiative, and Shockley-Read-Hall (SRH) recombinations.

## Results and Discussion

### Current Matching Process

Figure 1b shows the ultimate photocurrent as a function of NW diameters from 60 to 200 nm. One can find the photocurrents of the two subcells match each other closely at the NW diameters of 100 and 180 nm. For hetero-epitaxial growth of GaInP NWs on silicon substrate, larger diameter of NWs means more dislocations form at the interface of hetero-structure [13]. Hence, in the following electrical simulation, the radial *p*-*n* junction (core diameter of 60 nm, shell diameter (*D*) of 100 nm, the filling ratio (*D*/*P*) of 0.5, periodicity (*P*) of NW unit 200 nm) is chosen. Figure 1c shows the absorption of NWA with NW diameter of 100 nm and Si film under AM1.5G illumination. One can find that above 90 % incident light is absorbed by this NWA/Si substrate structure, which implies the high absorption capacity of the proposed two-junction solar cell. Furthermore, the aforementioned discussion also indicates that with fixed subcell thickness, the condition of current matching in the III–V NW/Si multi-junction can be fulfilled by adjusting the diameter of the NWs. Such an optimization process for current matching differs greatly from that in conventional multi-junction cells in which an anti-reflection coating design combined with subcell thickness adjustments should be used.

### Minority Carrier Lifetimes

**Fig. 2:**
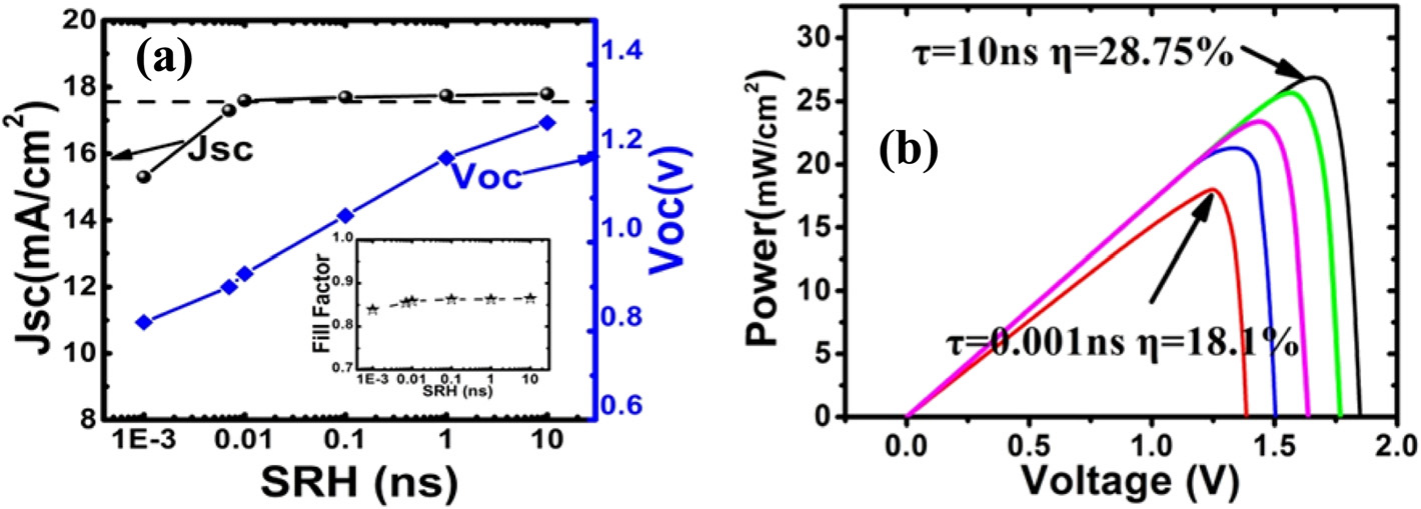
**a** The variation of simulated open-circuit voltage (*V*_oc_) and the short-circuit current (*J*_sc_) with *τ*_SRH_, *black dashed line* is the calculated *J*_sc_ of the bottom Si subcell, the *inset* in **a** shows the variation of fill factor (FF) with *τ*_SRH_. **b** Power-voltage characteristics of the III–V NWs/Si solar cells under one sun AM1.5G illumination at various *τ*_SRH_

The minority carrier diffusion length determines the PCE in such a way that it affects the photogenerated carrier collection and, hence, the short-circuit current (*J*_sc_) and the open-circuit voltage (*V*_oc_) and fill factor (FF). To examine the effects of the densities of mid-band gap trap state on the photogenerated carrier collection of the top NWA solar cell, we have performed simulations with SRH minority carrier lifetimes (*τ*_SRH_) ranging from 10^−12^ to 10^−8^ s, which reflects varying the densities of mid-band gap trap state. Figure 2a and its inset show the variation of simulated *V*_oc_ and the *J*_sc_ and FF with *τ*_SRH_. One can find *V*_oc_ decrease substantially from 1.28 to 0.81 V when *τ*_SRH_ varies from 10 ns to 1 ps. This result agrees well with that reported by Kayes et. al., who presented the degree to which the open-circuit voltage varied with the trap density depended most strongly on the trap density in the depletion region [14]. The FF was kept above 0.86 as *τ*_SRH_ > 7 × 10^−8^ s. In the case that *τ*_SRH_ = 1 × 10^−12^ s, the FF undergo only a tiny decrease to 0.839. This tiny drop may be attributed to series intrinsic resistance enhancement caused by high defect density under low minority carrier lifetimes. When the *τ*_SRH_ = 10 ns, the *J*_sc_ of the top core-shell *p*-*n* cell is 17.7 mA/cm^2^. This value is very close to the calculated photocurrent (18.2 mA/cm^2^) of this optimized structure as shown in Fig. 1b. Note that when the *τ*_SRH_ decreases to 0.01 ns, high short current (>17.5 mA/cm^2^) can be maintained. In this case, above 96 % photogenerated carrier can be effectively transported to the electrodes. These phenomena imply the high photogenerated carrier extraction capabilities of this radial *p*-*n* junctional structure. Considering that the electrical current matching must be satisfied for the series-connected cell structure, the character of radial NWA’s *J*_sc_ not sensitive to the bulk defects is very promising for fabricating two-junction solar cells. However, when *τ*_SRH_ is further decreased to 1 ps (the minority hole diffusion length was estimated to be 8 nm), the *J*_sc_ decreases greatly owing to the high photogenerated carrier recombination before reaching the built-in electric field region.

Figure 2b shows power of the proposed two-junction cell variation with voltage. The PCE can be found to decrease progressively with the reduction of *τ*_SRH_. Obviously, although current matching can be easily satisfied, relatively lower trap densities in top GaInP NWA is needed to obtain higher PCE of the proposed two-junction cell.

### Surface Recombination Velocity

The high surface-to-volume ratio represented by the adoption of a cylindrical radial junction increases both the active depletion region as well as the overall surface area of the device, which then becomes more susceptible to recombination of photogenerated charge carriers. In addition, the band bending arising from Fermi-level pinning at the surface of NW must also be taken into account in the electrical simulation. In view of this, we assume acceptor-like surface trap states, whose energy level lies near the middle of the energy band gap, are located at the surface of *n*-type GaInP shell layer. According to the surface recombination model given by Stevenson and Keyes [15], the recombination rate can be described as:

1 $$ {R}_{S,t}={N}_{\mathrm{trap}}\frac{\sigma_n{\sigma}_p{v}_n^{\mathrm{th}}{v}_p^{\mathrm{th}}\left({n}_s{p}_s-{n}_{i,\mathrm{eff}}^2\right)}{\sigma_n{v}_n^{\mathrm{th}}\left({n}_s+{n}_1\right)+{\sigma}_p{v}_p^{\mathrm{th}}\left({p}_s+{p}_1\right)} $$

where σ_*n*,*p*_ and ${\nu}_{n,p}^{\mathrm{th}}$ are respectively capture cross section and thermal velocity of electron and hole. To further simplify the recombination model, we assume that σ_*n*_ = σ_*p*_ = σ and ${\nu}_n^{\mathrm{th}}={\nu}_p^{\mathrm{th}}={\nu}^{\mathrm{th}}$. Meanwhile, when the semiconductor is exposed to the sunlight (far from equilibrium state), formula (1) can be simplified to:

2 $$ {R}_{S,t}={N}_{\mathrm{trap}}\sigma {v}^{\mathrm{th}}\frac{n_s{p}_s}{n_s+{p}_s} $$

For *n*-type semiconductor, *R*_*s*,*t*_ = *S*_*t*_*p*_*s*_ where SRV *S*_*t*_ = *N*_trap_*σv*^th^. In this way, one can correlate the density of surface trap states with SRV.

**Fig. 3:**
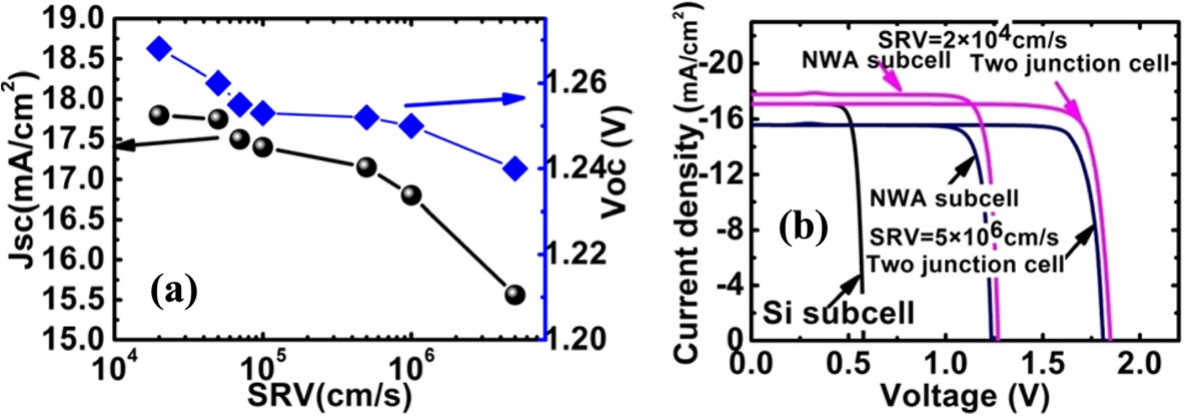
**a** The variation of *V*_oc_ and *J*_sc_ with SRV. **b** The *J*-*V* characteristic for the Si bottom cell, NW top cell, and series-connected two-junction cell at SRV = 2 × 10^4^ cm/s (after passivation) and 5 × 10^6^ cm/s

Figure 3a shows the variation of *V*_oc_ and *J*_sc_ with SRV. *V*_oc_ progressively decrease with larger SRV but is relatively not as insensitive as to *τ*_SRH_. However, when SRV increases from 0 to 5 × 10^6^ cm/s, *J*_sc_ decreases substantially from 17.78 to 15.56 mA/cm^2^. About 15 % photogenerated photocurrent annihilated, compared with the cell with perfect surface. Obviously, a large amount of photogenerated carriers recombine at the surface of NWA, due to the high surface-to-volume ratio of the proposed top NWA cells. Meanwhile, the electric field caused by band bending will drive minority carriers to migrate toward the surface. This in turn promotes surface recombination of minority carriers. Hence, in the top NWA subcell, the surface recombination is responsible for the annihilation of minority carriers and decides the *J*_sc_ of the proposed NWA cell. As expected, one can find that PCE of proposed two-junction cell decrease to 24.8 %, when SRV of the top GaInP cell increased to 5 × 10^6^ cm/s. This is because the decrease of photogenerated current in the top subcell will restrict the output current in the bottom Si active layer, considering current matching must be satisfied between the two series-connected subcells.

From the aforementioned discussion, to effectively extract the photogenerated carrier and achieve high efficiency of the proposed two-junction cell, it is necessary to control both the bulk and surface defect densities of the top NWA cell. In practical preparation, III–V NWs with low crystallographic defects can been successfully fabricated through carefully adjusting parameters of epitaxial growth techniques such as metal organic vapor-phase epitaxy (MOVPE) [16] and molecular beam epitaxy (MBE) [17]. Hence, the condition of high minority carrier lifetime can be obtained by the existing NW growth techniques. Meanwhile, for GaInP semiconductor, SRV value of 2 × 10^4^ cm/s can be obtained by (NH_4_)_2_S_*x*_ passivation treatment [18]. Note that this value is 2–3 factor lower than arsenic compound semiconductors such as AlGaAs and GaAs. In this case, the proposed cell yields a very promising device efficiency of 27.5 % with a short-circuit current density (*J*_sc_) of 17.38 mA/cm^2^, an open-circuit voltage of 1.84 V, and a fill factor of 0.86 under the illumination of one sun (AM1.5G).

## Conclusions

In conclusion, a device physics model incorporated with optical characteristics has been established to analyze the electrical performance of GaInP NWA/Si two-junction solar cell in term of its *J*-*V* characteristics and, hence, the PCE. From our simulation, it is suggested that the current matching of each subcell can be fulfilled by adjusting the diameter of the top NWA cell. Furthermore, as a photovoltaic device compromised by NWA, both SRH and SRV have great effect on the performance of the proposed solar cell. A promising PCE of 28.15 % can be obtained, considering the practical surface and bulk defects in GaInP semiconductor. This performance is comparable with that of state-of-the-art two-junction cell under one sun illumination (http://www.nrel.gov/news/press/2013/2226.html?print).

## Abbreviations

FDTD: Finite-difference time-domain
NWA: Nanowire array
P3HT: Poly(3-hexylthiophene)
PCE: Power conversion efficiency
PML: Perfect match layer
